# Development of Boron-Containing PVA-Based Cryogels with Controllable Boron Releasing Rate and Altered Influence on Osteoblasts

**DOI:** 10.3390/polym15071653

**Published:** 2023-03-27

**Authors:** Seda Ceylan, Ryan Dimmock, Ying Yang

**Affiliations:** 1 Department of Bioengineering, Faculty of Engineering, Adana Alparslan Türkeş Science and Technology University, Adana 01250, Turkey; sceylan@atu.edu.tr; 2 School of Pharmacy and Bioengineering, Keele University, Stoke on Trent ST4 7QB, UK; r.l.dimmock@keele.ac.uk

**Keywords:** borax, cryogel, degradable polymers, PVA, MG63, cell aggregation

## Abstract

Cryogel formation is an effective approach to produce porous scaffolds for tissue engineering. In this study, cryogelation was performed to produce boron-containing scaffolds for bone tissue engineering. A combination of the synthetic polymer, poly(vinyl alcohol) (PVA), and the natural polymers, chitosan and starch, was used to formulate the cryogels. Boron was used with a dual purpose: as an additive to alter gelation properties, and to exploit its bioactive effect since boron has been found to be involved in several metabolic pathways, including the promotion of bone growth. This project designs a fabrication protocol enabling the competition of both physical and chemical cross-linking reactions in the cryogels using different molecular weight PVA and borax content (boron source). Using a high ratio of high-molecular-weight PVA resulted in the cryogels exhibiting greater mechanical properties, a lower degradation rate (0.6–1.7% vs. 18–20%) and a higher borax content release (4.98 vs. 1.85, 1.08 nanomole) in contrast to their counterparts with low-molecular-weight PVA. The bioactive impacts of the released borax on cellular behaviour were investigated using MG63 cells seeded into the cryogel scaffolds. It was revealed that the borax-containing scaffolds and their extracts induced MG63 cell migration and the formation of nodule-like aggregates, whilst cryogel scaffolds without borax did not. Moreover, the degradation products of the scaffolds were analysed through the quantification of boron release by the curcumin assay. The impact on cellular response in a scratch assay confirmed that borax released by the scaffold into media (~0.4 mg/mL) induced bone cell migration, proliferation and aggregation. This study demonstrated that boron-containing three-dimensional PVA/starch–chitosan scaffolds can potentially be used within bone tissue engineering applications.

## 1. Introduction

Scaffolds are a key component in tissue engineering which aims to regenerate functional tissues through engineering approaches [1]. An ideal scaffold should have the ability to accommodate cells, support cell proliferation and desired differentiation, as well as promote cell migration, cellular attachment and vascularisation [2]. Methods including solution casting–salt-leaching, freeze–thawing, fibre binding and electrospinning are the most used techniques to produce porous scaffolds. Cryogelation methods which typically produce interconnected macroporous cryogel structures are relatively newly developed techniques. These methods require freezing solutions of monomers or polymers and then freeze-drying [3]. The unique properties of cryogels, being their chemical and mechanical stability and relatively simple fabrication process, make them potential candidates for tissue engineering scaffolds [4,5]. Cryogelation has begun to be the subject of studies in the last twenty years in the tissue engineering field [6]. The reason for this is related to the cryogels’ excellent supermacroporous interconnected pore morphology which creates a favourable environment for cells to penetrate and form new tissues [7]. The use of synthetic chemical crosslinkers is very common in the preparation of cryogels; however, it may decrease the biocompatibility of the scaffolds. Physical cross-linking methods can also be used; however, the stability and homogeneity of the cryogel scaffold may be compromised. Both methods have advantages and disadvantages which have important influence on the final characteristic properties of the scaffolds.

Great efforts have been made in the tissue engineering field to produce scaffolds with acceptable physical, chemical and mechanical properties. In addition to these properties, scaffolds should be biocompatible and accelerate tissue regeneration. To this end, both natural and synthetic polymers can be selected in combination because of their complementary mechanical properties, biocompatibility, degradation profile and regenerative capability in tissues [8]. Numerous studies have shown that natural polymers play an important role in cell-to-cell and cell-to-extracellular-matrix interactions due to their biocompatibility. Synthetic polymers are intensively utilised to imitate the native extracellular matrix mechanically [9]. The addition of growth factors, drugs, ceramics and metallic elements into scaffolds can play an important role in directing the migration, growth and the organisation of cells during tissue regeneration, as well as in the mechanical stabilisation of the defect site [10,11]. Other studies that combine natural/synthetic polymers and additives have shown remarkable benefits in tissue engineering constructs. Given this insight, various synthetic polymers have been explored in depth to obtain artificial scaffolds for bone tissue engineering applications [12,13,14,15,16]. The principal benefit to constructs by this approach is their crucially controllable material properties such as elasticity and degradability. These properties make synthetic polymers more beneficial in fabricating composites with desired functions [17]. On the other hand, natural macromolecules often include bioactive motifs which are critical for the cell matrix interaction and are ecologically sustainable [16,17].

Boron has been found to be involved in several metabolic pathways including ion transport, hormone production, calcium metabolism and bone growth [18,19]. It is known that there are approximately 230 kinds of boron minerals in nature [20,21]. Boron (3% boric acid solution) has previously been shown to increase the healing rate of deep wounds and decrease the duration of stay in intensive care [22]. In previous studies, the positive effect of boron on the osteogenic differentiation of the MC3T3-E1 pre-osteoblastic cell was shown by Gümüşderlioglu [23]. In a different study, it had been reported that the addition of boron accelerated the osteogenic and odontogenic differentiation potential of dental stem cells [24,25].

In this study, cryogelation method was utilized to produce scaffolds with poly(vinyl alcohol) as the synthetic polymer alongside starch and chitosan as natural polymers. Borax (sodium tetraborate decahydrate), the boron source, was incorporated with the dual purpose of a cross-linking modulating agent and a cellular stimulus. For this purpose, in the first part of the study, cryogel scaffolds were prepared using different ratios of PVA according to molecular weight and borax amount. The interaction effects of PVA molecules, high and low molecular weight and boron content on cryogels’ physical properties were investigated. Scaffold degradation products released into the medium were analysed by quantifying the boron amount. The non-destructive imaging modality, optical coherence tomography, was used to analyse the hydrated cryogels’ pore structure, whilst scanning electron microscopy examined the morphology of the dehydrated scaffolds at high magnification. The rheological properties and compression moduli of the cryogel scaffolds were measured. In the second part of the study, cryogel scaffolds with the adequate properties were selected and the cellular response to these cryogels was assessed with the MG63 human osteosarcoma cell line. The assessment of the cellular response comprised the cytocompatibility (CCK8) assay, cell migration (scratch assay), and cell aggregation with the confirmation of bone nodules (Alizarin Red assay). This project designed a new formula and fabrication protocol for boron-containing cryogel scaffolds and revealed an interesting interplay between these scaffold fabrication methods and their influences on bone cell activities in culture.

## 2. Materials and Methods

### 2.1. Preparation of Cryogels and Assessment of Their Cryogelation Capacity

The cryogels were made via the blending of PVA, starch and chitosan. PVA (99% hydrolysed), starch and chitosan (50,000–190,000 Da, 75–85% deacetylated) were purchased from Sigma-Aldrich, Gillingham, UK. The total polymer concentration of the PVA/starch/chitosan (P:S/C) blend solution was fixed at 3% (*w*/*v*). PVA with high molecular weight (Mw), 85,000–124,000 g mol^−1^ (H), and low molecular weight of 30,000–70,000 g mol^−1^ (L) was mixed in different ratios to produce cryogels (Table 1). The ratio of PVA and starch was fixed at 4:1 *w*/*w*. The PVA/starch mixture solution was placed in an autoclave chamber (JP Selecta, Med 20, Abrera, Spain) for an hour until reaching dissolution. Chitosan solution in the volume of 1% (*w*/*v*) was prepared in 3% acetic acid. The blends of PVA, starch and chitosan solution (P:S/C) were made through adding an equal volume of PVA/starch solution and chitosan solution, and then were stirred continuously at room temperature for approximately 20 min. The final polymer ratio in the solution was fixed at P:S/C—4:1:1, *w*/*v*. For cryogels which had borax crosslinker agent added (Sigma-Aldrich, UK), 30 mg/mL borax aqueous solution was prepared. Different concentrations of borax-containing (1, 2, 3, 4 and 5% (*w*/*w*)) solutions were formed through adding borax solution dropwise into the P:S/C solution under magnetic stirring. Finally, the mixtures, cast within in a mould, were transferred to the freezer at −20 °C and incubated for 24 h (Wisd Laboratory Instrument, Wertheim, Germany). The solutions without borax were produced and crosslinked only physically via freezing. After the 24 h incubation at −20 °C, all samples were freeze-dried for 24 h (FreeZone Benchtop Freeze DrySystem-7670531, Labconco, Missouri, USA) and the freezing dryer parameters were controlled at −60 °C at 0.1 atm.

**Figure 1:**
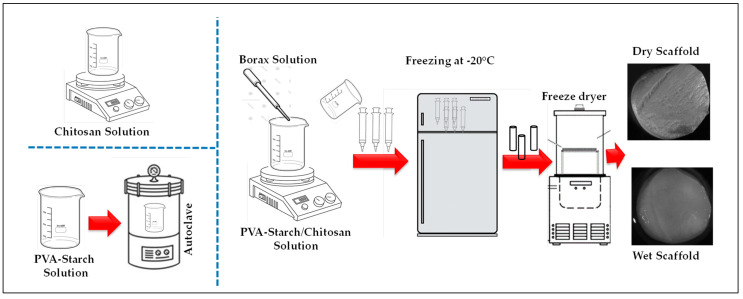
Schematic overview of the production steps for the P:S/C cryogel scaffolds.

Figure 1 shows the production steps of the cryogel scaffolds. Scaffold codes were written according to the PVA molecular weight, the ratio of high- and low-molecular-weight components and borax amount. For example, the specimen using the PVA with the 50:50 ratio of high Mw to low Mw (*w*/*w*) and 2% borax content was denoted as 50:50 2B.

### 2.2. Characterisation of the Cryogels

The effect of PVA molecular weight and borax content on the cryogelation capacity was assessed via the mechanical integrity of resulted cryogels by immersing them in water. Multiple mechanical, morphological and chemical assays have been undertaken.

#### 2.2.1. Pore Structure via Optical Coherence Tomography and Scanning Electron Microscopy

An optical coherence tomography (OCT) device was used to analyse the cryogels’ internal structure. OCT was performed using the spectral radar OCT device (Thorlabs, Ely, UK). Cross sectional B-scan images were taken using the LSM03 lens with lateral resolution of 13 µm and central wavelength of 1310 nm. The cryogels were fully hydrated and imaged whilst maintaining their hydrated state.

For scanning electron microscopy (SEM) measurement, the cryogels were presented in the dehydrated form. Hitatchi (TM4000 plus, Tokyo, Japan) and the associated software were employed to produce SEM images, using an accelerating voltage of 15 kV under medium vacuum utilising the secondary electron (SE) detector. The samples were sputtered with a gold coating ~10 nm thick prior to imaging.

#### 2.2.2. Degradation Rate

In vitro degradation studies were performed by incubating pre-weighed cryogel specimens in phosphate-buffered saline (PBS) in an incubator at 37 °C for up to 6 weeks. The specimens were dried and weighed each week. Each value was the average of the result of three parallel measurements. After that, the degradation rates of scaffolds were calculated by the following equation:

DR(%) = [(Wi – Wf)/Wi] × 100(1)

where Wi was the initial dry weight of a cryogel, Wf was the dry weight of a cryogel in the sampling week and DR was the degradation rate.

#### 2.2.3. Rheology and Compression Tests

Rheology was performed using the Kinexus Pro+ instrument (Netzsch, Selb, Germany). The cryogels were analysed in the fully hydrated state. A stepped frequency table oscillation program was used to test the gels at a frequency ranging from 0.1 Hz to 10 Hz, applying a shear strain of 0.5%. Temperature was maintained at 25 °C, and flat untextured geometries were used throughout the series.

The Testometric mechanical testing system (X250-2.5, Rochdale, UK) was employed for compression testing in a customised compression figuration. A steel piston of diameter ~13 mm was attached magnetically to the underside of the upper compression plate, whilst a non-slip PDMS surface of stiffness an order of magnitude greater in comparison to the test matter was laid upon the base plate. The 250 Kgf load cell was fitted to the upper compression plate. Compression was applied at a rate of 0.1 mm/s, and the test for each was aborted after exceeding the linear region (>0.100 N force applied). The test was conducted under constrained condition. A solid polystyrene compression cup was fabricated to contain the cryogels, with the wall internal diameter being slightly larger than the specimen diameter, allowing the test piston to contact the cryogels but not permitting further lateral expansion.

#### 2.2.4. Boron Content in Degradation Solution

The curcumin assay was used to determine boron content released from scaffolds into the degradation solution after the given incubation period. The assay consisted of multiple steps and the details are shown in the established protocol [26]. Firstly, 300 µL of the liquid specimen was transferred to an eppendorf tube and 100 µL 0.1 N HCl acid solution was added and mixed using a vortex mixer. Secondly, 50 µL extraction solution (2-ethyl-1,3-hexanediol 10% (*v*/*v*) in chloroform) was added and mixed well using a vortex mixer. Samples were centrifuged at 14500 rpm for 5 min. Fifty µL of the lower organic phase was taken and transferred to a new eppendorf. Afterwards, 200 µL acid mixture (sulphuric acid (conc.) and acetic acid (conc.) 1:1 (*v*/*v*)) was added to the lower organic phase and mixed well using a vortex mixer. Two hundred and fifty µL of 2 mg/mL curcumin solution in methyl-isobutyl-ketone was added to the mixture and mixed. Finally, after 1 h incubation, 500 µL of Milli-Q water was added to stop the reaction. Two hundred µL of upper phase solution was taken to measure the absorption using a spectrophotometer (Bio Tek/Agilent, California, USA) at 550 nm.

### 2.3. Cellular Response to the Cryogels

#### 2.3.1. Cytotoxicity Tests

Cytotoxicity assays were performed with osteosarcoma (MG63) cells. The MG63 cell line was purchased from ATCC^®^, Teddington, UK. The culture medium was low-glucose Dulbecco’s-modified Eagle’s medium (DMEM, Lonza, Verviers, Belgium) supplemented with 10% foetal bovine serum (FBS, Lonza) and 1% penicillin/streptomycin. Cultures were maintained in a humidified incubator (NUAIRE, Minnesota, USA) at 37 °C, in which the CO_2_ level was kept constant at 5%. P:S/C cryogels were sterilised in 70% ethanol solution, and then cryogels were washed thrice with PBS before the study. The P:S/C cryogels were cut into cylinders of a diameter of 9 mm and thickness of 2 mm. In total, 5 × 10^4^ MG63 cells in 50 µL medium were seeded on top of each cryogel and the control groups (wells without cryogel). This was the optimal seeding media volume used to prevent cell loss from the bottom of the scaffolds during the initial attachment duration (4 h). After this duration, the cell laden scaffolds were transferred to a fresh well plate, ensuring that subsequent cellular attachment on the bottom of the wells during the culture period were a result of migrated cells from the scaffolds, not unbound cells during seeding. These cryogels were placed in each well of a 24-well plate. Control and cryogel groups were cultured at 37 °C in a humidified 5% CO_2_ incubator with the medium changed every 3 days up to 11 days.

After the incubation period, growth medium from each well was removed and 10% CCK8 reagent in media was added to each well and incubated for 2 h. The colour change in the reaction mixture was detected in a spectrophotometer by measuring the absorbance at 460 nm. The CCK8 assay was used to analyse cell viability after 1, 3 and 7 days (d).

#### 2.3.2. Cell Migration

MG63 cells were seeded following the same procedure as stated in 2.3.1. The specimens were used to observe cell migration from the scaffolds using an optical microscope (Olympus, CKX414, Southend-on-Sea, UK). At different culture time points, cellular images were acquired and compared for the scaffolds which had different composition and borax concentrations.

#### 2.3.3. Alizarin Red Stain

After 11 days in culture, the cell-seeded scaffolds were transferred to another 24-well plate. The cells remaining in the bottom of the wells were washed twice with PBS and fixed with 4% paraformaldehyde for 20 min, then washed with PBS (×3) for 10 min. To estimate the calcium deposits of the MG63 cells migrating from the cryogel scaffolds, Alizarin Red staining solution was added to each well and incubated for 20 min; then, the staining solution was removed and washed with deionised water (×5) to completely remove the unbound stain. The stained cells were observed under an inverted microscope (Leica, Milton Keynes, UK).

#### 2.3.4. Scratch Assay

The spreading and migration capabilities of cells in response to boron molecules in the culture media were assessed using a scratch wound assay. The MG63 cells were seeded on 48-well tissue culture plates and cultured for 48 h at 37 °C in culture medium to nearly confluent cell monolayers. A linear wound was generated by using a sterile 200 μL plastic pipette tip to scratch the cell monolayer with defined width. The cellular debris was removed by washing the wells with PBS. 

Five groups of borax-containing media were used. DMEM media supplemented (control group) with borax (0.01 and 0.005 mg/mL) were produced as positive control groups. Alongside the positive control groups, the culture media from scaffolds with different released borax concentrations (due to scaffold degradation and released unbound borax) were used in the scratch assay. After one-week incubation, the media from scaffolds with variable PVA Mw ratios (100:0, 70:30 and 50:50) and fixed amounts of borax (3%) were used. Three representative images of the scratched areas from each condition were taken to monitor cell migration. The studies were performed in triplicate per group and incubation was maintained up to 48 h incubation.

The scratch assay was used to verify the cell migration assay in boron-containing solution. In the cell migration experiments, Section 2.3.2, cells were seeded on the scaffolds, and the boron release was continuous and increasing in the media during the culture, whilst scratch assay generated insights into cell migration and proliferation due to the presence of the scratch. The difference in using degradation products of the scaffolds for the scratch assay was that the boron concentrations in the culture media were known or fixed; this is a direct contrast to the migration and proliferation of cells in situ with the presence of scaffolds.

### 2.4. Statistical Analysis

Statistical analysis was performed with Origin Lab Pro (2019). One-way ANOVA and Tukey were performed to determine significance for the assay. *p* < 0.05 was considered to be statistically significant.

## 3. Results and Discussion

### 3.1. Fabrication Feasibility of P:S/C Boron-Based Cryogels

The first goal of this study was to investigate the effect of different molecular weights of PVA and borax concentration on the cryogelation capacity and associated microarchitecture of the P:S/C cryogel scaffolds. PVA was chosen for this study because of its hydrogel formation capacity and good mechanical properties. The first natural polymer used was starch, which has the key characteristics of being biodegradable and one of the most abundant and inexpensive polysaccharides. The second natural polymer was chitosan, which consists of glucosamine and N-acetyl glucosamine. Chitosan is a natural cationic polysaccharide, with non-toxic, biocompatible and antibacterial properties [27,28].

In this study, we investigated how PVA molecular weight in the blends and the concentration of the chemical cross-linking agent, borax, affected the cryogelation and the key properties of the cryogel scaffolds. Table 1 summarises the appearance and integrity of the formed cryogels with different PVA composition and borax concentration.

It was shown that the high-molecular-weight PVA strongly influenced the cryogelation capacity. The produced cryogels with high-molecular-weight PVA alone (100:0) or with the ratio up to 50 (50:50) exhibited an intact cryogel appearance for borax amounts of 1, 2, 3, 4 and 5%. However, 4–5% borax addition led to rubbery-textured cryogels. In contrast, the formula with low-Mw PVA (0:100) could not produce stable scaffolds even by adding borax up to 5%. From this study, it was observed that borax amount and molecular weight of the PVA affected the cryogel qualities through different mechanisms. One of the outcomes was that adding 5% borax content (*w*/*w*) led to unstable products. In addition to this, using only low-molecular-weight PVA caused unstable products. The reason for this may be due to the short chain length in low molecular PVA. Solely short-chain cryogels cannot produce polymer networks with a high density of cross-linking, which would enable the formation of stable and strong cryogels. The physical cross-linking reaction through hydrogen bonding was dominated by the molecular weight of PVA. Chemical cross-linking occurs between borate ions and the OH groups in the polymer chains of PVA. High-molecular-weight PVA has long chains and low-molecular-weight PVA has short chains. Interestingly, high borax content enhanced PVA formulations with a high ratio of low molecular weight, for example, 30:70 groups. In the groups without borax or low borax contents (30:70 0B, 30:70 1B and 2B), weak cryogels formed. When borax content increased to 4–5%, the 30:70 4B and 30:70 5B formed intact cryogels.

To summarise the production and selection processes of appropriate scaffolds for further investigations, we present evidence that both physical cross-linking and chemical cross-linking can affect the cryogelation capacity. When high-molecular-weight PVA was the dominant component, borax-induced chemical cross-linking did not exert much influence upon the properties of the final product, but it enhanced the cryogelation capacity for the groups with specific ratios of a low-molecular-weight PVA component, and the chemical cross-linking increased the molecule length or cross-linking density.

### 3.2. Degradation Rate

**Figure 2:**
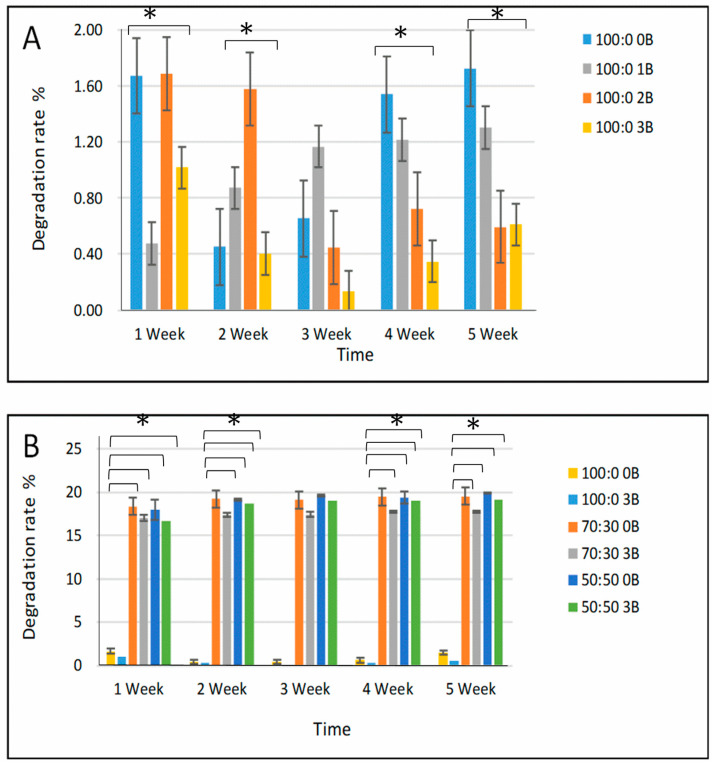
Degradation rate of different cryogel scaffolds up to 5 weeks incubation. (**A**) The cryogels with only high-Mw PVA and variable borax contents. (**B**) The cryogels with mixed Mw PVA and variable borax content. Significance was determined by ANOVA test (* *p* < 0.05).

We selected two series of cryogels for comparative degradation studies: one was cryogels using a high-Mw PVA but with varied borax content as shown in Figure 2A, and another group comprised of the cryogels using varying ratios of Mw of PVA but a fixed borax content at 3%. The cryogel without borax was used as the control. It was revealed that all cryogels degraded. A mass loss between 1.7% and 0.6% was observed for the cryogels which were produced only using a high Mw of PVA. Seemingly, increasing the borax content in the cryogels reduced the degradation rate, indicating that the additional chemical cross-linking played a role in the final products and stabilised the products.

As presented in Figure 2B, the weight loss of cryogels which included low-Mw PVA was significantly higher than the groups in the high-Mw PVA group. This weight loss was observed to increase generally with increasing low Mw of PVA percentage in cryogel formulation. The low degradation ratio of cryogels in Figure 2A may be attributed to the high Mw of PVA and borax which correlates to dense polymer network formation and small pore size distribution in cryogels. These characteristics are proposed to reduce the hydration rate and cleaving rate of chemical bonds.

Two different mechanisms influenced the degradation rate of these cryogels through PVA’s Mw and borax amount. The degradation rate of the scaffolds without borax increased from 1.2% to 18.3% as the high Mw of PVA amount decreased from 100:0 to 50:50 (Figure 2B). Conversely, with the same Mw content of PVA but a different amount of borax 50:50 0B and 50:50 3B, a reduced degradation rate (19.12% versus 18.68%) was observed. This result confirmed that borate ion can make chemical cross-linking bonds with the -OH groups in the PVA chains, which prevented degradation.

In different studies, the degradation profile of the scaffolds was examined via enzymatic digestion in contrast to this study, wherein PBS was kept as the degradation solvent. Chitosan can be degraded in lysozyme solution and starch can be degraded in amylase solution. Baran et al. incubated chitosan and starch composite membranes in enzyme solutions of alpha-amylase, lysozyme and PBS separately [29]. However, there are no enzymes in the human body that can digest PVA and PVA cannot be hydrolytically cleaved. The degradation mechanism of PVA consists of physical erosion and dissolution [30,31]. The solubility of PVA in water depends on its degree of polymerisation and degree of hydrolysis. As a general principle, the intra- and inter-molecular hydrogen bonding controls the water solubility of the polymer [32]. The presented cryogels in this study have a high mass ratio of PVA; hence, the degradation study of PBS reflected the main degradation behaviour of the cryogels because PVA dominated the mass of the cryogels.

### 3.3. Pore Structure Observed Using OCT and SEM

**Figure 3:**
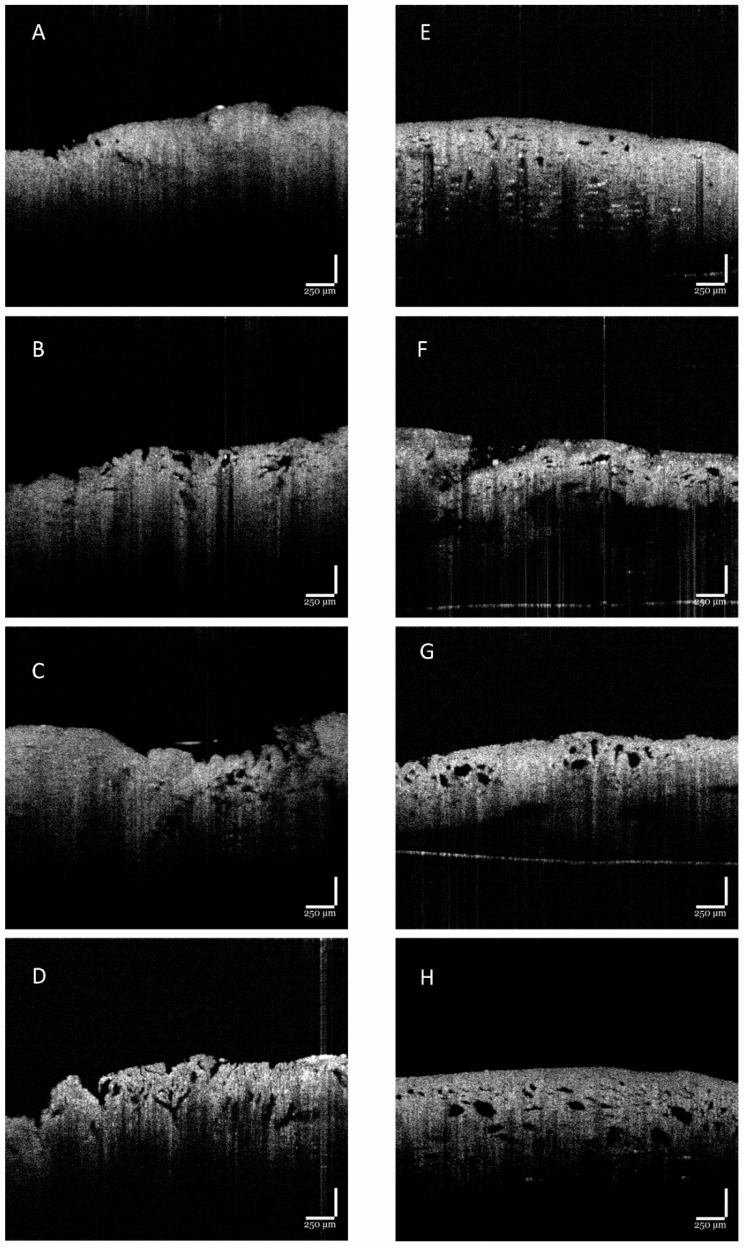
OCT images of cryogel scaffolds: (**A**) 100:0 0B, (**B**) 100:0 1B, (**C**) 100:0 2B, (**D**) 100:0 3B, (**E**) 50:50 0B, (**F**) 50:50 1B, (**G**) 50:50 2B, and (**H**) 50:50 3B. Scale bar is 250 µm.

**Figure 4:**
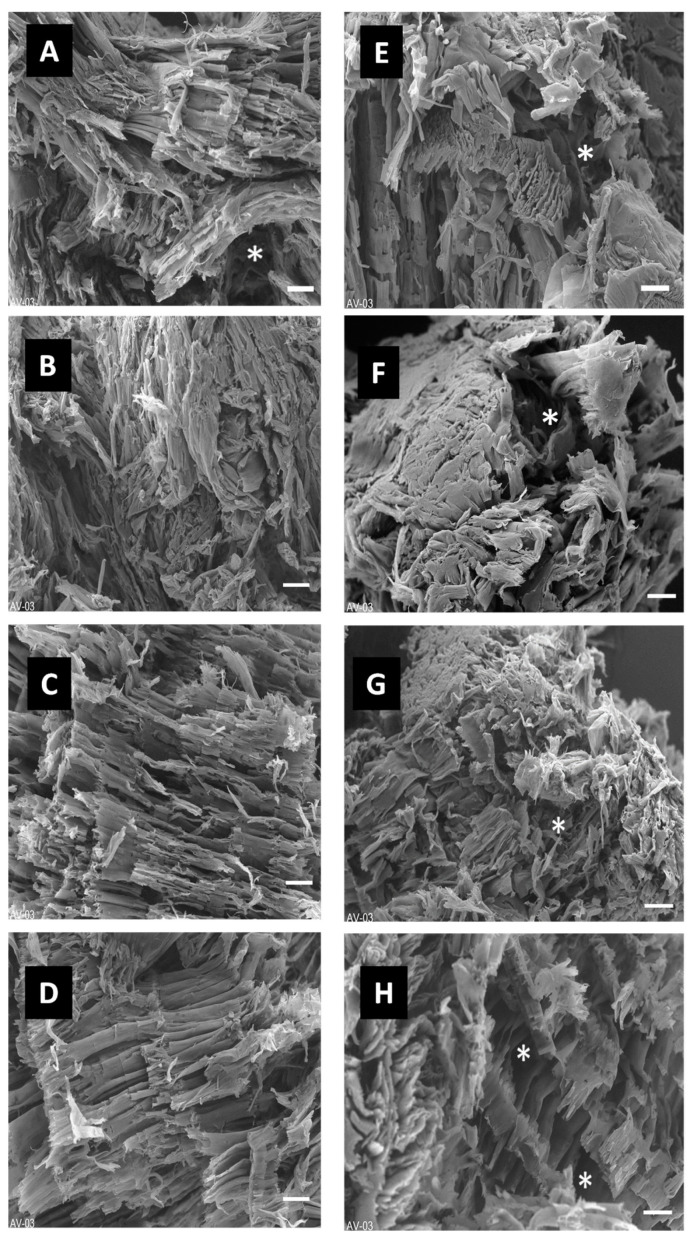
SEM images of cryogel scaffolds: (**A**) 100:0 0B, (**B**) 100:0 1B, (**C**) 100:0 2B, (**D**) 100:0 3B, (**E**) 50:50 0B, (**F**) 50:50 1B, (**G**) 50:50 2B, and (**H**) 50:50 3B. Scale bars are equal to 100 µm. * indicates the macro-pores.

The morphology of PVA/starch–chitosan-based scaffolds and the dispersion of pores in the scaffolds were evaluated using optical coherence tomography (OCT) and scanning electron microscopy (SEM) (Figure 3 and Figure 4). In principle, the freeze-drying and cryogelation procedure resulted in interconnected porous morphology in cryogels. When changing PVA molecular weight and adding the chemical cross-linking agent, borax, the cryogels gained macro-pores.

The cryogels using solely high-Mw PVA exhibited a dense appearance, particularly the 100:0 0B scaffold group. Apparently, the addition of borax did not considerably change the smoothness and density of the cryogel architecture. Visually, macropore presentation increased with borax concentration.

The density obviously decreased as the ratio of high Mw of PVA amount decreased from 100:0 to 50:50, as shown in Figure 3. Interestingly, the macro-pores appeared in all 50:50 groups. The pores were relatively homogeneous across the thickness of the cryogels. Comparing the effect of borax agent on the pore morphology of cryogels from Figure 4, it was also observed that in the cryogels with borax addition, the higher borax content group had a high presence of macro-pores. This significant difference in pore size and density might be ascribed to the cross-linking reaction and network density due to the difference in Mw of PVA and borax content, thus resulting in heterogeneous strain distribution upon rehydration. The cryogels without borax and only high-Mw PVA consisted of a small porous structure. Mixed (50:50) Mw PVA-based cryogels had pores that were quite large and interconnected through thin walls. This formulation permitted the fast transportation of water molecules across thin walls over short distances through the macroporous structure and affected the change in the degradation profile. Overall, assessments from OCT confirmed that the 100:0 series had low macro-pore density and the pore size was below the 50 µm region, whilst the 50:50 series had macro-pores up to the 120 µm region.

The SEM images verified the OCT images of both series of the cryogels. In the dehydrated state, the 100:0 series showed long, collimated and densely packed fibrous morphology with low macro-pores, whilst the 50:50 series exhibited short, randomly orientated fibrous morphology with macro-pores. In the lower borax content samples, cavities caused pits, and in the higher borax content samples, cavities appeared as cross-planar fractures across fibrils.

### 3.4. Rheological Assessment and Compression Test Results

**Figure 5:**
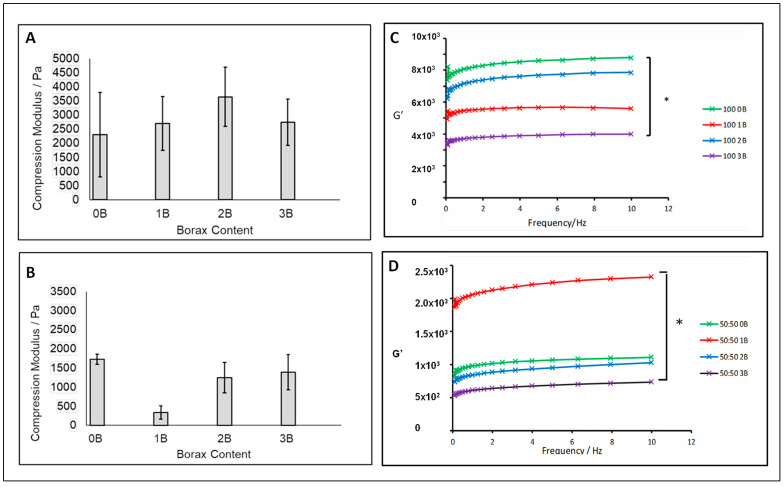
Compression results (**A**,**B**) and the frequency dependence of storage modulus (G′) (**C**,**D**) for cryogel scaffolds. (**A**,**C**) The cryogels with only high-Mw PVA and variable borax contents; (**B**,**D**) the cryogels with 50% Mw PVA and variable borax contents (significance was determined via ANOVA (* *p* < 0.05)).

Two cryogel groups were selected to assess their compression and rheological properties, as shown in Figure 5. The first group was the cryogels with high-Mw PVA but with varied borax content, 100:0 0–3B, and the second group was the cryogels with mixed PVA Mw (50:50) but varied borax content, 50:50 0–3B.

The mechanical properties of the cryogels were evaluated via compression testing and the mean values of compression plots are presented in Figure 5A,B. The three investigated variables, the Mw of PVA, the percentage of the used Mw of PVA and borax concentration, had different effects on changing the cryogel compression moduli. By lowering the ratio of high-Mw PVA, the cryogels became soft and flexible. However, the compression modulus value did not demonstrate a significant difference upon varying the amount of borax (*p* > 0.05). The 50:50 1B group showed high heterogeneity and a low modulus compared to other groups. The underlying mechanism for the low value is not fully understood yet, which requires future investigation.

In general, the storage modulus of cryogels in 100:0 0–3B series were higher than those in 50:50 0–3B. Furthermore, in the 100:0 0–3B series, the group without borax addition showed the highest storage modulus (G′), whilst the 100:0 2B group gained a higher modulus value than 100:0 1B and 100:0 3B. The modulus change pattern in the 50:50 0–3B series was different from the 100:0 0–3B series. The 50:50 1B group demonstrated a much higher storage modulus than the other groups, whilst 50:50 3B had the lowest G′ value. The different G′ values in these two series confirmed that PVA molecular weight dictated the mechanical property of the cryogels. The chemical cross-linking via the borax and -OH group in PVA can alter the mechanical properties depending on the PVA molecular weight and borax concentration. Higher Mw PVA prevented the effective reaction with borax due to its fast reaction rate and low mobility; hence, the increase in mechanical strength through the borax reaction (G′ 100:0 0B vs. G′ 100:0 2B) was limited. Low Mw PVA in 50:50 0–3B series promoted the borax reaction with PVA due to the high mobility, hence resulting in a large increase in the mechanical strength and G′ of 50:50 1B in comparison to G′ of 50:50 0B.

### 3.5. Boron Content in Degradation Solution

We assessed the boron content in the degradation solution with the aim of correlating the amount of free borax (trapped in the network) and the chemically bound borax under different reaction conditions. In this study, the determination of boron content was undertaken using the curcumin assay [26]. Colorimetric-based assays using curcumin can be used easily for the routine analysis of boron. A spectrophotometer was used to measure absorbance at 550 nm. Table 2 shows the amount of boron released in a one-week culture medium from different cryogel scaffolds. Utilising the borax chemical formula, boron content (nmole) was derived by using the established protocol [26].

The results show that changing the PVA molecular weight in the scaffold affected the degradation rate and borax release. In this study, the borax amount was fixed at 3% to analyse the effect of the Mw of PVA on the scaffolds. From Table 2, it can be observed that decreasing the content of high Mw of PVA in scaffolds resulted in decreased boron content in degradation products. This can be explained by the changes in the cross-linking degree in scaffolds with borax because of the Mw of the PVA. Spoljaric et al. showed that borax readily crosslinks PVA via “di-diol” complexation [33]. Lower Mw PVA has shorter molecular chains and higher mobility than high-molecular-weight PVA; hence, borax could attain a high reaction rate with -OH groups of PVA. As a result, the majority of the borax was chemically bonded to the network and the un-crosslinked amount of borax was less in the 50:50 3B scaffold than in 100:0 3B. The curcumin assay analysis confirmed that 100:0 3B cryogels retained more free borax, and thus released more borax when incubated compared with the 50:50 3B scaffold. Although the boron release was only monitored for one week, it is common that burst release in the first few days leads to the highest concentration of included small molecules. Hence, the data of boron in the culture media for the first one week of culture would present the highest boron content among prolonged cultures. The pilot data have paved the way for a good understanding of the cytotoxic effect of the scaffolds in long-term culture.

### 3.6. Biological Response of the Cryogels

#### 3.6.1. Cytotoxicity (CCK-8 Assay)

**Figure 6:**
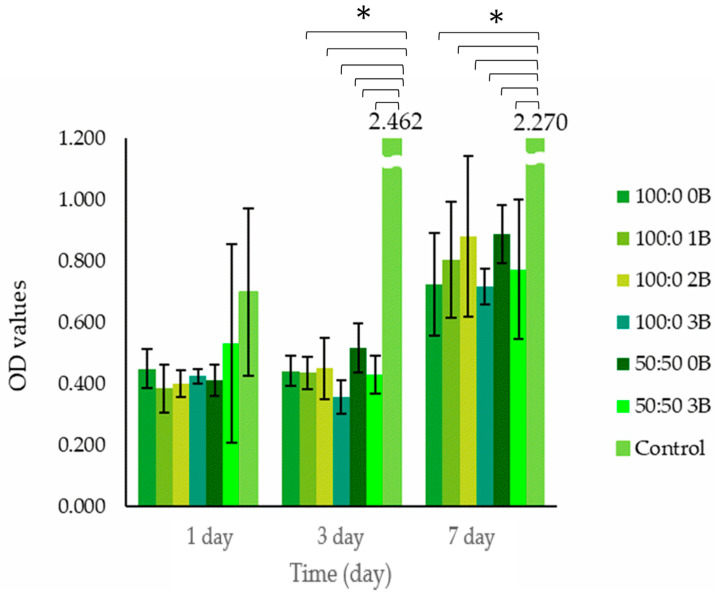
Cell viability of MG63 cells seeded on cryogel scaffolds and cultured up to 7 days, assessed via CCK-8 assay (significance was determined via ANOVA test (* *p* < 0.05)).

The CCK-8 assay results for MG63 cells seeded in the cryogel scaffolds after 1, 3 and 7 days in culture are presented in Figure 6.

In comparison to the control (cells on tissue culture plastic plate), the cell number was lower in the scaffolds at all three time points. The marginally lower O.D values in the cryogel groups compared to those in the control group on Day 1 suggests that there might be cell loss during the initial cell seeding period (the estimated cell loading efficiency was 75%). The estimation for loading efficiency was accounted for the cryogels’ highly porous nature. Some cell suspension could have been lost as free cells into the culture container. The much lower O.D value on Day 3 and Day 7 in the cryogel groups might be due to the slow cell proliferation rate in scaffolds. However, there was not much difference in O.D value across the different composition groups, implying that all cryogel scaffolds were sufficiently biocompatible to support living cells. It was revealed that a slightly lower cell number/metabolic rate was demonstrated in 3% boron-containing scaffolds.

#### 3.6.2. Induction of Bone Nodule Formation

Inspired by the physical characterisation and boron content assay results on the cryogels, we hypothesised that the added borax compound in the cryogel formula was not incorporated into the cryogels completely through chemical cross-linking. Some of the borax molecules were trapped in the cryogels and were easily released as an active boron source. This active source was able to affect cellular metabolism either directly on the scaffolds or induce cell migration and subsequent aggregation through the free borax molecules in the incubation media. Demirci et al. produced and analysed poly-(lactide-co-glycolide) (PLGA) scaffolds containing boron (sodium pentaborate) for bone tissue engineering applications. In this study, different amounts of sodium pentaborate pentahydrate were added and in vivo assessments were performed after 28 days of implantation. The results showed that scaffolds with a high concentration of sodium pentaborate pentahydrate increased bone formation. Boron (3% boric acid solution) has been shown to increase the healing rate of deep wounds and decrease the duration of stay in intensive care facilities [21].

**Figure 7:**
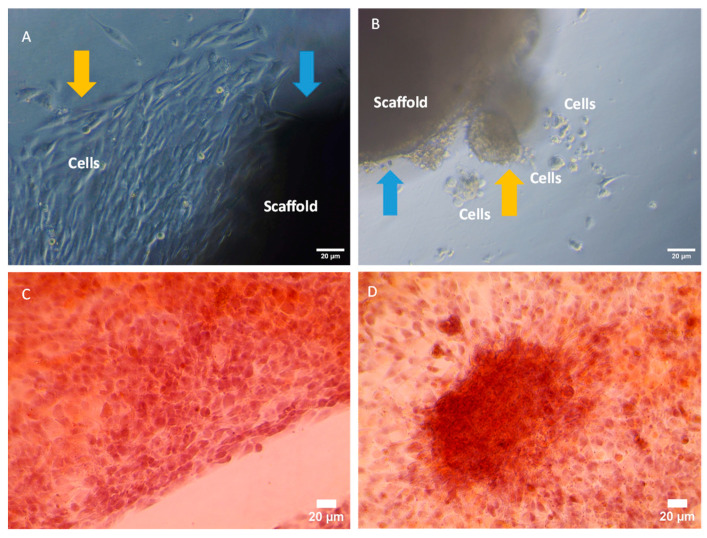
(**A**,**B**) Live images of cell migration from cryogel scaffolds after 7 day culture; (**C**,**D**) Alizarin Red staining images of the migrated cells after 11 day culture. (**A**) 100:0 0B, (**B**) 100:0 3B, (**C**) 100:0 0B, (**D**) 100:0 3B. Blue arrows indicate scaffolds; yellow arrows indicate migrated cells.

In the first assay, we used high-Mw PVA groups with different borax content, and 100:0 0B versus 100:0 3B. In total, 5 × 10^4^ MG63 cells were seeded onto each cryogel with a dimension of ϕ 9 × 2 mm. After 7 days in culture, the cells migrated from the scaffolds. Different cell morphologies are clearly demonstrated in Figure 7.

The cells that migrated from the 100:0 0B cryogel exhibited a typical elongated spindle shape of the MG63 cell line in normal monolayer culture; however, the cells that migrated from 100:0 3B were aggregated (Figure 7A,B, day 7 images). The staining with Alizarin Red dye for the cells with 11 days of culture showed that the aggregates had dark red staining, suggesting the aggregates might be bone nodules with high calcium content, whilst the cells from the 100:0 0B group only exhibited background staining. The cells in the normal monolayer culture without the cryogels exhibited the same background staining (data not shown). The results were consistent with a previous report on boron’s effect on bone formation. Nagarajan et al. [34] used fused deposition modelling (FDM)-based 3D printing to produce biomimetic biodegradable scaffolds made of polylactic acid (PLA). They strengthened these scaffolds by adding exfoliated boron nitride (EBN) as a filler component. Mineralisation (i.e., calcium deposition on scaffolds) was determined using colorimetric quantification after incubation with the Alizarin Red-S dye following MG-63 cell culture on the 3D printed scaffolds for 1, 14 and 21 days. The results showed that the deposition of calcium on Day 21 was remarkably higher in cultures where cells were grown on PLA/EBN compared to on PLA scaffolds, demonstrating good mineralisation on scaffolds. These results demonstrated that the addition of exfoliated boron nitride was biocompatible and showed good bioactivity by enhancing MG-63 cells [34]. Boric acid also affects the mineralisation degree of bone cells and a study showed that after Days 15 and 21, a significant increase in ALP activity was seen only in the 6 ng/mL-treated rat bone marrow mesenchymal stem cells (BMSCs) compared to the control and 6 µg/mL-treated cells [35].

In the second study, we performed a scratch assay as a convenient tool to further reveal the relation between the free borax content in cryogels and the cryogel formula. It was predicted that a high boron content would kill the MG63 cells, but the appropriate boron content would promote bone cell differentiation. Three cryogel groups with different PVA molecular ratios of 100:0, 70:30 and 50:00, but at fixed borax content of 3%, were selected. The media with a given concentration of borax, 0.005 mg/mL and 0.01 mg/mL, were included for the comparison. Table 3 shows the group arrangement and the media collection conditions.

**Figure 8:**
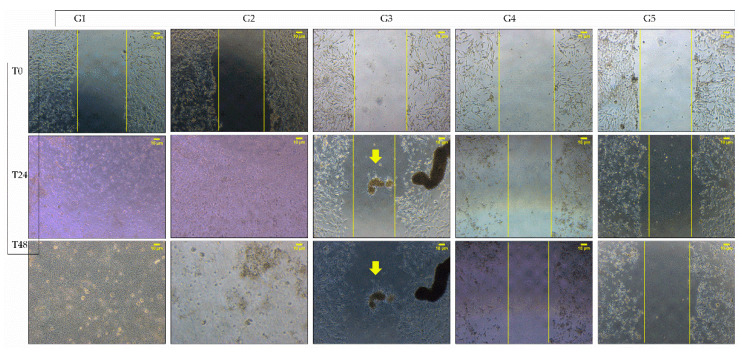
Bright field images of the MG63 scratch assay incubated for 24 h and 48 h in the defined borax content media, and the extracted media from 5 different groups. Group 1: extract from cryogel 100:0 3B; Group 2: extract from cryogel 70:30 3B; Group 3: extract from cryogel 50:50 3B; Group 4: 0.01 mg borax/mL media; Group 5: 0.005 mg borax/mL media. The yellow lines indicate the scratch boundary and cell migration. The arrows indicate the cell aggregates.

The bright field scratch assay images of MG63 cells after scratch formation at 0, 24 and 48 h are presented in Figure 8.

All groups had a clear cell monolayer scratch at T = 0. By culture time at 24 h, MG63 cells in Groups 1 and 2 were detached, and dead cells covered the scratching area with no live cell morphology remaining. The scratches on Groups 3, 4 and 5 remained visible, with Group 3 showing clear cell aggregation within the scratch and along the scratch boundary. Group 4 and 5 showed no cell migration. The similar cell morphology across the five groups remained on the 48 h culture with less cell entities in Groups 1 and 2, and a wider scratch distance in Groups 4 and 5. On the other hand, the wound closure rate was also calculated and Group 3 demonstrated the greatest closing area (Supplementary Data). In addition to these, the sizes of the aggregates in Group 3 increased. These scratch assay results correlated well with the curcumin assay. In the curcumin assay, it was revealed that 100:0 3B released 4.982 nmol (1.9 mg/mL) boron, 70:30 3B released 1.850 nmol (0.704 mg/mL) and 50:50 3B released 1.081 nmol (0.411 mg/mL). We estimate that when the borax concentration in the culture media was around 0.4 mg/mL, as in Group 3, cell migration and aggregation were induced. A borax concentration above 0.7 mg/mL killed cells, as in Groups 1 and 2. However, borax concentration in 0.05 mg/mL or below exerted no effect on bone cell migration and nodule formation.

Altogether, this study has generated a consistent protocol to tailor cryogel mechanical properties, porous morphology and bioactive boron release concentration, via the assessment of their cellular responses for bone formation. However, the study has limitations which must be addressed in the future, which will obtain more promising data for translational tissue engineering applications. For osteogenesic applications, further investigation of the degradation profile, utilising enzymatic conditions over a prolonged period, is required to better simulate in vivo conditions. The calcium-like tissue formation in bulk cryogel scaffolds should be investigated to determine the boron effect on osteogenesis as the form trapped within cryogel alongside free form in media. The expression of osteogenesis genes via PCR and other protein markers, such as ALPase and collagen type 1, should be investigated as well.

## 4. Conclusions

A novel bioactive scaffold for tissue engineering applications, which combines the three polymers PVA, starch and chitosan and the cross-linking agent borax, has been developed using cryogelation. It was revealed that the borax concentration and molecular weight of PVA were critical fabrication variables of cryogel production. In addition to this, the degradation profile and borax concentration in the incubation extract and the mechanical properties of scaffolds changed with these critical variables. The cryogels using a high ratio of high-molecular-weight PVA led to greater mechanical properties and a low degradation rate but less chemical incorporation of borax into the scaffolds, and hence higher borax content in the incubation media than their counterparts. With respect to the MG63 cell assays, the results indicate that borax-crosslinked P:S/C cryogels had good cytocompatibility for tissue engineering applications. Using the appropriate formula and fabrication methods could induce the cryogel scaffolds to release borax in the approximate amount of 0.4 mg/mL into media, which promoted bone cell migration, proliferation and the formation of bone-nodule-like aggregates. This study demonstrated that we can optimise the formula of P:S/C borax-containing cryogels and maximise the role of borax as a cross-linking agent and as a bioactive compound to promote bone differentiation during culture.

## Figures and Tables

**Table 1:** Effect of PVA molecular weight and ratio and borax % on cryogelation capacity.

PVA (%)		Borax Amount (%)					
H *	L *	0	1	2	3	4	5
100	0	Intact cryogel	Intact cryogel	Intact cryogel	Intact cryogel	Intact cryogel LR **	Intact cryogel LR
70	30	Intact cryogel	Intact cryogel	Intact cryogel	Intact cryogel	Intact cryogel LR	Intact cryogel LR
50	50	Intact cryogel	Intact cryogel	Intact cryogel	Intact cryogel	Intact cryogel LR	Intact cryogel LR
30	70	Weak cryogel	Weak cryogel	Weak cryogel	Intact cryogel	Intact cryogel	Intact cryogel
0	100	Dissolve in water	Dissolve in water	Dissolve in water	Dissolve in water	Dissolve in water	Dissolve in water

* H: high Mw, L: low Mw, ** LR: like rubber.

**Table 2:** Boron amount in one-week culture media (within the degradation product). The presented values are the mean of three replicate measurements.

Sample	Absorbance	Boron Amount (Nanomole)
100:0 3B	0.378	4.982
70:30 3B	0.150	1.850
50:50 3B	0.094	1.081

**Table 3:** Media contents of scratch assay for MG63 cells.

Groups	Ingredient
Group 1	100:0 3B% degradation product for one-week/2 mL media
Group 2	70:30 3B% degradation product for one-week/2 mL media
Group 3	50:50 3B% degradation product for one-week/2 mL media
Group 4	0.01 mg borax/mL (0.04 nmole boron)
Group 5	0.005 mg borax/mL (0.02 nmole boron)

## Data Availability

Not applicable.

## Supplementary Materials

The following supporting information can be downloaded at: https://www.mdpi.com/article/10.3390/polym15071653/s1, Table S1. Wound closure rate from scratch assay; Figure S1. Representative fluorescence image of MG63 cells seeded on 100:0 3B cryogel scaffolds for 3 days culture through Live Dead assay. Green: Live, Red: Dead cells. Majority of cells were live and homogenously distributed with small portion dead cells.
