# Supplementary material for: Development of Boron-Containing PVA-Based Cryogels with Controllable Boron Releasing Rate and Altered Influence on Osteoblasts
Source: Polymers (Basel). 2023 Mar 27;15(7):1653. doi: 10.3390/polym15071653 (PMC10097227; doi:10.3390/polym15071653)
Supplement: Supplementary file 1 [file polymers-15-01653-s001.zip › polymers-2207441-supplementary.pdf]

<u>SUPPLEMENTARY MATERIAL</u>

Article

Development of Boron-Containing PVA-Based Cryogels with Controllable Boron Releasing Rate and Altered Influence on Osteoblasts

Seda Ceylan [1,2], Ryan Dimmock [2] and Ying Yang [2,*]

[1] Department of Bioengineering, Faculty of Engineering, Adana Alparslan Türkeş Science and Technology University, Adana, Turkey; sceylan@atu.edu.tr
[2] School of Pharmacy and Bioengineering, Keele University, Stoke on Trent, ST4 7QB UK; r.l.dimmock@keele.ac.uk
* Correspondence: y.yang@keele.ac.uk; Tel.: 00441782674386

Wound Closure Calculation

Wound closure rate was calculated using Imagej programme. The area was calculated by using T0 and T24 and T48 time point and the results showed that the group 3 demonstrated clear cells aggregation within the scratch and along the scratch boundary. Group 4 and 5 showed no cell migration and closure rate was not linear or stable.

Supplementary Material 1: Table S1

Table S1. Wound closure rate from scratch assay

	Scratch Area μm2					Wound Closure Ratio %				
	G1	G2	G3	G4	G5	%G1	%G2	% G3	%G4	%G5
T0	12.873.169	12.561.824	13.206.063	14.979.648	14.184.046	can not be calculated	can not be calculated	2.683.941.459	6.275.033.966	9.661.770.696
T24			12.851.62	14.039.67	12.813.616	can not be calculated	can not be calculated	1.404.182.508	3.423.627.478	-3.390.924.154
T42			11.047.018	13.559.004	13.248.116					

Supplementary Material 2: Figure S1

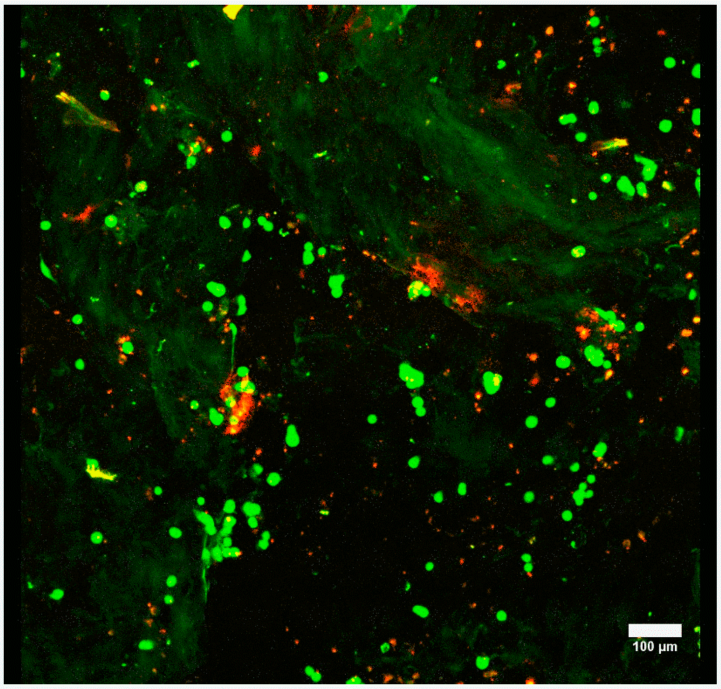

Figure S1. Representative fluorescence image of MG63 cells seeded on 100:0 3B cryogel scaffolds for 3 days culture through Live Dead assay. Green: Live, Red: Dead cells. Majority of cells were live and homogenously distributed with small portion dead cells.
